# Bright’s Disease of the Kidneys

**Published:** 1860-11

**Authors:** 


### NORTH AMERICAN

### MEDICO-CHIRURGICAL REVIEW.

NOVEMBER, 1860.

### Analytical and Critical Reviews.

ART. I.—*Die Brightsche Nieren-krankheit und deren Behandlung. Eine Monographie.* Von DR. FRIED. THEOD. FRERICHS, etc.

*Bright's Disease of the Kidneys, and its Treatment. A Monograph.* By DR. FREDERICK THEODORE FRERICHS, Ordinary Professor of Medicine, and Superior of the Medical Clinic in Kiel. With One Copper-plate. Brunswick: F. Vieweg & Son, 1851. pp. 286.

DR. FRERICHS'S treatise is one of those fine, complete, and well-rounded-off monographs which do so much honor to the German schools, and which are so much wanted, at least in a larger supply, among the Anglo-Saxons of both sides of the water. When a large amount of medical inquiry has been expended on a subject in great part new, few things contribute more to human improvement in the case, than a strict and honest abstract of all that is ascertained, divested of matter of parade, and omitting nothing really useful. Not only is this the case as regards the practitioner at the time, but for the service of him who wishes to extend medical science. From the work issued, a new start is taken. As Emerson, the mathematician, roughly remarks, "a man can easily see a great deal farther when mounted upon the shoulders of another;" he who has mastered a well-digested system can the more readily concentrate his industry upon special portions of the history which call for further development and examination; and science advances by a process demanding a less outlay of labor upon the examination of many volumes, at the risk, after all, of making unfortunate omissions. To do this, it is necessary that the abstract should be honest and complete, dictated by a love of truth and science, and not of display. To this category fully and unquestionably belongs the work before us. What a contrast to that ambition which would make science consist of a series of revolutions, usurp the labors of the past without acknowledging the obligation, labor to give limited or long-known things the aspect of fundamental novelties, and toil to shake the confidence of patients and students in what is already known, rather than to add valuable matter to the amount!

Of 286 pages, small octavo, filled with matter so rich as to defy condensation, we omit the preface. This is occupied with a view of the present state of medical inquiry, and the special reasons which led the author to the choice of this subject. To the latter we may add the frequency of the disease in the vicinity of his clinic.

In medical history, Dr. Richard Bright, of London, enjoys a peculiar glory; as the description of the disease in a great measure begins with him, little having been collected from previous authors, ancient or modern, yielding more than detached or vague indications of what was left for him to combine, compare, and render more exact and useful. Still the progress of knowledge seems, in approaching this, to have followed its usual laws, and prepared the way for the coming event. This required the agency of the man, equally inclined to make all use of the observations and comments of those who preceded him, and not to neglect attending to the wants of the sick and his own opportunities of better understanding what he saw.

Hippocrates and Galen express themselves on the subject in a manner entirely indefinite. "But where the kidneys cannot 'draw,' they cause the display of a sudden dropsical condition." Aëtius knew that hardening of the kidneys was accompanied with anasarca. Avicenna indicates the same thing; and adds, that in this state of things the urine is thin and small in quantity. Toward the end of the sixteenth century, openings of diseased bodies became more frequent, and the results were applied more rationally to the solution of pathological questions. Observations of maladies of the kidneys which appeared to be the groundwork of dropsical affections, became more numerous. Schenk, Bonetus, Lieutaud, and particularly Morgagni, describe cases which, with greater or less exactness, represent Bright's degeneration, including one or two of the more serious and remarkable of the symptoms.

At a much later period, the alterations which the urine undergoes in this condition of things received adequate attention. The examinations of the urine made by the older physicians were almost always confined to simple inspection; by which the presence or absence of substances of easy solubility, as albumen, etc., could not be determined.

Cotugno was the first who proved by experiment the presence of albumen in hydropic urine. Unfortunately, he misunderstood his experiment; imagining that the albuminous matter arose from the dropsical fluids passing into the urinary cavities, and took the process for an effort of nature at a cure. Cruikshank first distinguished more accurately: he knew that albumen was present in the urine only in special kinds of dropsy. Our subject was investigated with far more accuracy and care by Wells, in the "Transactions of a Society for the Improvement," etc. He succeeded in indicating and establishing the almost constant presence of blood or albumen in the dropsy which succeeds scarlet fever; and that, at least in many cases, pain in the region of the kidneys and material alterations in the parenchyma of those organs accompanied the coagulable character of the urine. Shortly after, in 1813, appeared Blackall's treatise on Dropsy; in which he adheres to the distinction, previously raised by Cruikshank and Wells, between dropsy with and without coagulable urine, as a principle of classification; and this he illustrates by a series of cases. Yet Blackall ascribed the coagulability of the urine less to a condition of the kidneys, and much more to a general inflammatory disposition of the whole organization; in contrast with other forms of dropsy proceeding from local alterations confined to any viscus, as the liver, heart, etc. The albuminous contents of the urine were valid to him as an indication for venesection, and not as a sign of the degeneration of the kidneys.

Thus stood the matter when Bright published his justly celebrated labors in 1827, 1836, and 1840, and "procured for himself, although late, the general gratitude of physicians." The connection between dropsy and disease of the kidneys, on the one hand, and the presence of albumen in the urine during many forms of dropsy, on the other, were known in the general outlines of the facts; and a relation between disease of the kidneys and coagulability of the urine was at least indicated; but yet this was neither pointed out with precision nor extended to its theoretical and practical consequences. Bright acquired for himself the merit of first proving the internal connection of an explicitly characterized affection of the kidneys with albuminuria and dropsy, and pointing out the frequent occurrence of this combination of symptoms, which, from the present time forward, must be regarded as a special form of disease. He followed up and described the anatomical basis of the malady in its manifold and changing forms, portrayed in a masterly manner its essential and accidental symptoms, firmly established the diagnosis, and brought to light the most important causal impulses active in its production. Bostock, at the same time, attended to the needful chemical investigations, described the condition of the urine, and gave indications of the changes which take place simultaneously in the blood.

The new history became the object of much discussion. Christison and

Gregory extended the developments of Dr. Bright, and confuted the intimations of doubt by Elliotson, Copland, and particulary by Graves. "A prophet is not without honor save in his own country;" and the remark has often been made, that the reputation of Dr. Bright in Great Britain bore no proportion to the esteem and respect in which he was held on the Continent, and most of all in Germany. Christison distinguished himself as a partisan and fellow-laborer of the native genius, by establishing with accuracy the relations of the fluids, and pointing out the distinctive characters of the acute and chronic forms.

Our space will not allow us to enumerate the various recent writers, in many instances of great merit, on this disease, now become an open object for public criticism. The terseness of Dr. Frerichs's diction has compelled us to copy many of his words. We proceed to his account of the morbid anatomy.

He alleges that the descriptions of the cadaveric appearances given even by the best authors have not been made from a complete and coordinated system, explaining the manner in which the diseased appearances are produced, by a thorough comparison with each other and with the natural structure; and that writers have contented themselves with enumerating the different organic changes as so many species or varieties of the effects of the disease. He justly illustrates the imperfection of this method by imagining what would have been the result had the same procedure been adopted with regard to pneumonia or tubercular consumption, instead of seeking for the causes of the variety of forms encountered in the nature of the processes which had previously gone on in the part. If we understand him correctly, he claims to have been the first who arranged the anatomical phenomena of Bright's disease with a due reference to this causative and consecutive method.

The Malpighian body, according to him, is a cluster of loops of fine arteries, inclosed in a capsule of nearly spherical form. The orifice seems to have little regularity in its direction. In his pretty wood-cuts, it generally turns to one side; its zigzag tube of escape turning downward, after many windings in the cortex, to the nearest pyramid and calyx. The arterial twig which enters the corpuscle is accompanied by a corresponding vein; which, on its escape from the capsule, immediately joins a net-work of corresponding vessels, covering the adjacent capsules in a very intricate manner. These, Mr. Bowman has called, from comparison, "the portal system of the kidneys." They, of course, all terminate in the renal vein.

The structure permits a very large amount of blood to pass, with great rapidity, through the organ; and in doing this, to be pressed with very great force, in comparison with other arterial currents, against the sides of the little vessels; a point of importance to be recollected. The wide and almost straight arteries of the kidneys enable the blood to be impelled, with almost the whole force of the contractions of the heart, against the sides of the small arteries in the capsule. By the minuteness and intricate coils of these last, the resistance of considerable friction is offered to the escape of the vital fluid into the veins. According to the measurement of Mr. Mogk, in order to cause the blood to flow from the renal veins with an equable stream, he found it necessary to apply the pressure of a column of blood in a glass tube two metres in height. Poiseuille expelled the fluids through the same route by the pressure of 1835 millimetres of water.

It is natural to believe that the quantity of fluid that exudes through the sides of the capillary loops into the calyces, under this heavy pressure, might be very considerable. With all the recent researches into the cause of the selection of particular substances in the separation of this or any other secretion, it remains still unknown, and we shall not risk an opinion on the probability of our ever knowing better. With the modifying mechanical and chemical circumstances the case is different. The composition of the urine, when first secreted, must depend, according to Dr. Frerichs, upon the amount of the hydrostatic pressure within these vessels, and upon the nature, elasticity, and size of the pores of their lining membrane. Whether this composition is afterwards modified, within the tubes, by further exhalation, or by absorption, is also unproved. We may assume it for a certain point, that, in the natural state, no albumen, fibrin, or fat ever escapes by this route. For this fact, in addition to general experience, he cites the investigations made by Matteucci, Cima, and Brücke, on the diffusion of substances through animal membranes. Besides the modifying agents which we have enumerated, it is probable that much influence is exerted by mechanical packing of the veins, and by altered innervation of the capillaries, through direct or indirect routes. Under these circumstances, first albumen and then fibrin escape; and it is not necessary for the coats of the capillaries to be burst, and for blood to issue from the rupture.

The delicate clusters of looped vessels in the Malpighian capsules are the first and principal portion of the vascular apparatus that suffers in this manner; and, in some instances, the remaining capillary system of the kidneys escapes the change. These are principally the cases which arise from impediments to the circulation; mainly structural imperfections of the heart. Where the whole capillary system of the kidney is affected, the case generally arises from disordered innervation. It is in the coils of the Malpighian bodies that the heightened pressure of the column of circulating blood first takes effect; and this is productive of the effusions.

Fibrin seldom reaches the ureters in a liquid form, but generally coagulates in the earlier urinary passages; principally in the flexuous and involved tubes of the cortical substance. Of these tubes, they present manifest moulds or impressions, which are visible on examination of the muddy sediment of the urine. They are expelled in large numbers by the urine secreted in the capsules; and carry with them the scales of the epithelial lining of the tubes.

In other cases the coagula remain longer in place, and undergo various changes. The epithelial scales, being macerated in the exudation, become muddy in their appearance, are filled with fat globules, and finally decay into a fatty detritus, or shrink into irregular masses. If the coagula remain still longer in the canals, the secretion gradually ceases on the part of the glomeruli; from the impulsive force of the blood being equaled by the mechanical resistance of the coagula, which fill up the secretory tubes, adhere to their linings, and do not escape. Coagulation takes place within the capillaries, their cavities are effaced, the external surface of the glomeruli is covered with a finely granular coating, and they are at last obliterated. In consequence of the progressive formation of fibrinous coagula, and their separation and discharge, many urinary canals remain more or less completely deprived of their epithelium, gradually collapse, become atrophic, and finally disappear. Others remain filled with crumbling, fatty exudation, and become increased in size; and sections of these, on incision of the organ, stand out from the atrophied vicinity in the form of little tubercle-like masses, of sizes from that of millet-seed to that of hemp-seed, and are known as granulations. The volume of the kidney, which, as long as so great a portion of the urinary tubes was distended with the matter of exudation, appeared increased, gradually shrinks with the disappearance of the canals, and becomes atrophied.

If a portion of the fibrinous exudation is deposited in the interstitial textures, an occurrence which seldom takes place in considerable amounts, it either crumbles, so far as it has not been absorbed in a fluid condition, into a finely granular detritus, or it more or less completely organizes itself as connective tissue, (cellular membrane,) which then partly surrounds the Malpighian corpuscles in concentric strata, and partly forms a net-work between the urinary canals.

From these anatomical processes, Dr. Frerichs deduces three stages of the disease, in each of which exists a material difference of the morbid changes, and also of the symptoms.

1. The stadium of hyperæmia, with commencing exudation.
2. That of exudation and of the commencing changes of the exudation.
3. That of retrogradation of development, and atrophy.

*First stadium.* The kidney is enlarged, its volume and weight not unfrequently doubled; or the latter increased from three to four, eight, ten, or even twelve ounces. The surface appears smooth; the tunica propria is muddy, red, and injected, and easily torn from its attachments; and the superficial venous net-work distended and protuberant with black blood.

The principal seat of the enlargement is in the cortical substance. This is more or less of a dark, brownish red, is brittle and easily torn, and emits, from sections by the knife, a viscid, bloody fluid, with which the parenchyma is infiltrated. On the surface, and in deeper sections of the cortical substance, are found dark-red spots of a roundish or irregular form, which give the kidney a speckled appearance. The pyramids, which contribute but little to the increase in volume, are also hyperæmic; and the redness has a streaky appearance, from the distribution of the vessels. The calyces and pelvis ordinarily contain a turbid fluid, most generally tinged with blood, and their lining membrane is tumid and coated with vascular arborescences. The more intimate texture of the kidneys, independently of hyperæmia, exhibits no perceptible lesion. The venous plexuses of the cortical substance project from distention with dark blood (meaning, of course, in dissections or dilacerations.) In like manner, the coils of vessels in the Malpighian capsules are more visible and projecting than ordinary, from distention with blood. The capillaries Dr. Frerichs has not found widened, though repeatedly measured, of course micrometrically, and the circumference of the capsules did not exceed the normal average. Hemorrhagic effusions are frequently found, sometimes from the glomeruli, sometimes from the vascular net-work which surrounds the urinary canals, and sometimes, in fine, from the superficial veins of the cortical portion. The capsules and tubes are found fully distended with blood; and on the surface of the organ, the most regular of the round spots arise from over-distention and bursting of the superficial coils of these tubes. In hemorrhage from the vascular net-work, apoplectic centres of greater or less extent are found, which sometimes render the tubes impassable. The superficial veins furnish flat extravasations beneath the tunica propria, which penetrate the substance below to a greater or less depth.

The epithelial lining, in this stage, is not necessarily altered. The coagula are generally found distending the canals of the cortical substance; more rarely of the pyramids; and are generally best exhibited by submitting the fluid expressed from incisions to the microscope. Sometimes they are simple fibrinous moulds of the canals; pale, homogeneous, transparent cylinders; sometimes there are a few scales imbedded in them, and sometimes they exhibit blood corpuscles, more or less altered, scattered singly or in thick groups; and sometimes the canals are seen with their lining entire, sometimes deprived of more or less of it.

The above description is principally taken from acute and tumultuous cases, following scarlatina, or severe exposures of the body to cold. In the chronic form of the disease these changes proceed more quietly; but they are seldom subjected to anatomical inspection. The enlargement of the kidney is considerably less; and often not noticeable. That hemorrhages frequently occur, is evinced, on the one hand, by the mixture of blood with the urine, and, on the other, by the remains of apoplectic effusions, found on examining such cases when advanced to their later stages.

The diagnosis of this stadium is often difficult; and it may be confused with simple hyperæmia. A criterion on which we may rely, is the appearance of fibrinous or bloody coagula, forming moulds of the canals. When these are found, the diagnosis is practicable in strongly-marked cases; but slighter grades are generally overlooked.

The number of instances in which the first stage is subjected to examination is small. Of two hundred and ninety-two cases of individuals who died of this disease, the first stage was observed in only twenty.

Bright, Rayer, and Martin Solon, according to our author, viewed this disease in its real character. Johnson considers it as a desquamative nephritis, the most explicitly in contradiction with fatty degeneration, of which he makes a distinct form of disease. In this opinion Dr. Frerichs does not coincide; and he believes it more rational and conformable to the harmony of science to explain the fatty product as being, in most instances, a product of the changes which take place in the exudation, which are to be described hereafter.

In regard to the bloody specks, there is more variety of opinion. Rayer, Becquerel, and Rokitansky take them to be violently-injected Malpighian corpuscles. Our author deems it incredible that these bodies are dilated to the one-tenth of an inch. Bowman, and afterwards Goodsir and Johnson, showed that the effect was produced by the distention with blood of coiled and interwoven vessels. Toynbee believed he had shown that the Malpighian capsules were distended to this size, and gives a figure; but Bowman and our author think him in error.

*Second stadium*, or that of exudation and of commencing metamorphosis of the matter which exudes. The hyperæmic condition of the vessels abates; and the exudative process gains the upper hand. According to the causes of the disease, and the degree of violence of the circulatory derangement, the effusion is sometimes confined to the urinary tubes, and sometimes takes place in the interstitial substance of the kidneys, particularly in the cortical portion. Later begins the metamorphosis. While, within the tubes, the epithelial matter and fibrin together crumble into molecules, containing fat globules, in rarer cases, they organize themselves, and become converted into connective membrane, round the Malpighian capsules, and between the tubes. The kidney gradually loses its redness of injection, and becomes of a dull yellow. At first, streaks and stellated spots are still seen; later, they disappear. The volume of the organ remains increased, sometimes more than in the preceding stage; and its weight may reach six, twelve, or fifteen ounces; most frequently so when the exudation escapes into the interstitial texture. The kidney becomes soft and brittle, and a muddy, milky fluid escapes from sections. In these it can be seen that the increased bulk is derived principally from infiltration of the cortical substance. This descends deeply, and not unfrequently indents the bases of the pyramids, separates the tubuli from one another, and gives the cones an unraveled and tufted appearance. The thickness of the cortical part increases to eight or ten lines, and sometimes to an inch. (French.) Section of the cortical substance exhibits a pale, yellow color, in which we can here and there distinguish completely yellow, insulated spots of a ramified or roundish form. The pyramids strongly contrast with the pale, cortical matter, by their more or less dark red, which is only interrupted by insulated brighter streaks.

Injection of the kidneys, in this stadium, succeeds but very incompletely. The calyces and pelvis have their mucous membrane swollen, and of a smutty red.

Histological inquiry exhibits changes far greater than in the previous stage. The capsules, in part, retain their customary size; but the glomeruli are less conspicuous within them from being covered with fine granular matter. Contact with acetic acid makes this last transparent; and the coils of vessels are more directly seen. It is not uncommon to meet with them quite empty of blood. Another portion of the capsules are visibly enlarged. In these, between the glomerulus and the capsule, lies a thick coating of solid fibrinous exudation, of a granular character, and mixed with numerous fat drops. In some cases, crystals of cholesterine occur in them. Some of the vascular loops still contain blood, sometimes fluid, and capable of being impelled forward by pressure, sometimes in appearance coagulated, and of a dull, dirty-brown color. The observation of Simon, that the vascular coil lies all crowded together and flattened, in the fundus of the capsule, is seldom verified. Near the diseased capsules it is always common to find others unaltered in their apparent structure.

Few of the canals in the epithelial portion entirely escape alteration in the epithelial scales. In common, the lining stratum becomes denser and more resisting; but is filled with granular matter, abounding in fat drops. When this has made further progress, the cells lose their polyhedral shape, and become rounded, changing the most remarkably where the natural adhesion is the closest. Part of them change gradually into granular masses, subsequently decomposed into detritus. This process is essentially the same which Reinhardt describes in the mammary gland during pregnancy, in pneumonia, in the Graafian vesicles, in the epithelium, in fine, of all organs affected with hyperæmia and the process of exudation. Other epithelial scales are always found in the vicinity, which simply wither without infiltration, and are converted into a similar detritus, coarser or finer; and this may even choke up the tubes. Portions of tubes are found, which, in place of epithelium or its scales, exhibit nothing but stratified fat globules.

Another portion of the tubes exhibits homogeneous fibrinous coagula; some recent and colorless, others older and yellowish. The fibrinous masses are structureless, and contain copious fat globules, and sometimes blood-disks, some of their ordinary appearance, others shrunk and shriveled.

The canals are in part visibly dilated even to double or triple their ordinary dimensions, and acquire projecting sinuosities, interrupted by contractions, thus presenting an appearance of varicosity. Sections have the aspect of a congeries of round or oval cysts, of from one-fiftieth to one-twentieth of a line in diameter. Nuclei are often found within them, sometimes thinly dispersed, at others in rows or thick masses, covering the walls of the tubes. At other times, insulated, roundish cells appear; and many tubes contain merely a homogeneous fibrin, or a fine granular matter mixed with fat globules; while there is sometimes the appearance of an empty space in the middle, lined, as in the engraving given, with a thick coat of fibrin.

Professor Frerichs thinks Mr. Simon in error when he sets down the formation of cysts as an essential part of the alterations of structure in Bright's disease; and believes that the appearance we have just described has given rise to the error. Cysts, when they do occur, are ascribed by our author to the monstrous development of epithelial cells, which are liberated by the destruction of the tunica propria of the urinary canals, and are not extruded by the exudation.

The different combinations and relative degrees of intensity of these changes are too numerous to be catalogued. The increase of size and weight varies from fifteen ounces to very little more than the customary healthy amount. In general, acute and dyscratic cases furnish much greater increase in size, and chronic and indolent ones, particularly from impeded venous circulation, very little. In the latter category, the alterations are very liable to be overlooked, and imperatively require the microscope; it being indispensable to identify the fibrinous contents of the urinary tubes. The danger of error is the greater, as those kidneys which are not much increased in size are not much changed in color and other external peculiarities, the extent of these depending on the amount of exudation. Similar variety is produced by the successive changes in the matter which has exuded; and a very advanced stage in this alteration furnishes the "yellowish granular matter" of various English writers. This frequently becomes conspicuous in those Bright's granulations which project from the surface of the undivided kidney, which are composed, as we have seen, of masses of distended urinary vessels, and not of enlarged Malpighian capsules.

Mr. Frerichs found evidence of the second stadium of the disease in one hundred and thirty-nine dissections out of a totality of two hundred and ninety-two.

The principal appearances of the second stadium are well characterized and represented in plates by the earlier writers. They classified the differences in extent and progress of the various alterations as distinct forms of the disease; which Mr. Frerichs recapitulates, with references. Particular praise is given to Messrs. Bright, Rayer, Christison, Martin Solon, and Rokitansky. The accounts of the Malpighian corpuscles are various; but the results harmonize with the views of our author. Bowman, Johnson, Todd, and Henle did not find these bodies altered, and doubted the fact of this occurring. Johnson believed that their contents escaped by rupture; but this Mr. Frerichs believes he has shown to be impossible. Later observers, as Canstatt, Siebold, Toynbee, and Hessling, have agreed in the description which our author has verified, and which we have given above. Toynbee describes the afferent vessels and the glomeruli of the capsules as having been found by him enlarged to a size greatly exceeding that recorded by other observers. Mr. Frerichs thinks he may have mistaken urinary vessels, distended with blood or with matter of injection, for arterial capillaries.

The discrepancies with regard to the urinary tubules are not important; and principally refer to the constructions to be put on facts, and not to the facts themselves.

*Third stadium*, of decomposition, or retrograde development and atrophy. We want a good English word to express the first of these ideas. "Invelopment," as contrasted with "development," which has been proposed, seems to us hardly enough characterized by habitual use or striking analogy, to prevent misapprehension or confusion in its employment. It is established by popular habit in a different meaning. So is "deformation," which contrasts with "formation." "Unmaking" has the best authority among old authors, but would be thought wanting in the dignity of science. Perhaps the best is "involution," which can be compared with "evolution."

The changes found in the third stadium are characterized by a more striking excess and variety; and more frequently startle the anatomical observer. It is among them, too, that the famous "Bright's granulations," so often spoken of as if they were the essence of the disease, are most numerous and conspicuous. The withering and great deformities which attract so much attention are of this stage.

A greater or smaller portion of the tissues of the kidneys disappears or is atrophied. The urinary canals, robbed of their epithelium, collapse; and at the same time the Malpighian corpuscles become crumpled together and shrunk. The basal membrane of the canals remains, but compressed into folds. In other cases, of rarer occurrence, where the effusion has largely taken place into the texture of the kidneys, the matter effused becomes organized into common connective (cellular) tissue, and afterwards undergoes the contraction of a *cicatrix,* forcibly compressing the Malpighian capsules and portions of urinary vessels around which it is effused.

The kidney frequently subsides to its customary dimensions, and often much below them; sometimes to the weight of three, two, or even one and a half ounces. The capsule of the organ is usually of a muddy-white color, and thickened in patches. It adheres firmly to the cortical substance, and is torn from it with difficulty, rarely without a portion of the substance of the kidney being separated with it; and often drags with it deep appendages, which leave furrows dividing the organ into lobes. The surface has lost its smoothness; and is uneven, and studded with minute tuberculiform projections. The granular protrusions on the surface are generally about the size of poppy-seeds or the heads of pins; and rarely, of hemp-seed or small peas. With the capsule in situ, the color of the organ is generally muddy yellow; the cicatricular retractions, commonly pale, in some parts bluish-black, from old extravasation of blood; but most frequently portions of organ are found which retain the natural brown color.

The kidney has lost its original brittleness; which is replaced by a leathery toughness that increases as the atrophy proceeds.

Sections, in general, exhibit a more or less considerable wasting of the cortical matter; this forming, in far-advanced cases, a mere border, of a few lines in width, round the bases of the pyramids. In other cases, where the exudation has been remarkably great, it may equal or exceed its natural size. In the thickness of the cortical matter, the same granulations as on the surface are still encountered, separated from each other by a pale, cicatricular tissue.

The pyramids are diminished in size, particularly at their base; but less atrophied than the cortex. Granulations are found interspersed between the straight vessels, near the base. The calyces seem wider, and their mucous membrane is puffy and of a grayish blue, and sometimes streaked with varicose vessels. The fatty mass round the kidneys increases with the wasting of the organ which it embraces.

Injections succeed very imperfectly, as many of the capillaries are impermeable; but dilated vascular branches, filled with dark blood, are not uncommonly seen upon the surface.

The progressive changes undergone by the exudation, and the atrophy of the urinary tubes and Malpighian capsules, are all apparent under the microscope. The tubes are either filled with the granular matter resulting from the decomposition which took place in the preceding stadium, or have partly emptied themselves. Well-marked epithelial scales, unaltered, or simply infiltrated with fat drops, are rarer; so are fibrous coagula. The principal contents of the irregularly protruding clusters of tubes are a uniform mass of protein granules and fat drops, but little altered by acetic acid. The atrophy of the tubes, in its various forms, meets the eye in the contracted spaces, in the puckered cicatrixes, in the parts surrounding the granulations, and in the deeper furrows produced by tearing. In these, the canals are nearly empty, collapsed, wrinkled and irregular in their outline, resembling an obscurely-fibrous mass. Still later, the fibrous character is only to be demonstrated by tearing the substance apart with the needle. They are to be here identified by a rigid outline, angular inflexions, and crumpled folds. Among them have been encountered distinct fibre cells, resembling those which produce smooth muscular fibre, and incapable of being discriminated from those found in the stroma of the healthy kidney.

Of the capsules, a few retain their natural size and appearance. Others are reduced to two-thirds or half their volume, and form globules or oval vesicles, more or less filled with fat drops. In detached instances, one-third of the capsule is filled with dark-colored spots, composed of crowded fat molecules. The vascular coils are buried beneath these matters, and difficult of detection. When the glomerule is exposed, by tearing the capsule, or pressing it to bursting, and then washing away the fat, it exhibits nothing more than a few pale, empty vessels, scarcely to be distinguished as such; and very often all search for the remains of this body is unavailing.

Where exudation matter occurs in the texture of the parts, and compresses the intervening points by contraction, besides the above changes, newly-formed connective or cellular substance is encountered. This is, in general, principally composed of elongated fibre cells, intermixed with which are fully developed fibrils; and both of them can be insulated and distinguished with certainty from the basal membrane of the canals. The capsules are enveloped in a coat of these, usually from one-seventieth to one one-hundredth of a line in thickness. Very commonly a portion of this matter is forced within the capsule, pressing upon the vascular loops, and insinuating itself between them. The tubes are closely embraced by the new connective membrane; and the intervening spaces can be shown by sections, of which the transverse answer best. In the larger intervals is sometimes seen a mixed mass, composed of the ruins of a number of urinary tubes, the capillaries of the interstitial tissue, and the newly-formed connective membrane, seemingly dissolved together in confusion; and sometimes fat drops are imbedded in them, singly or in rows.

The milder cases, in which the kidney is not remarkably altered in size, are frequently overlooked, especially by inexperienced observers. The changes in color and consistency are not calculated to strike the attention, and the granulations are little developed. From these to the most deformed kidneys, occur a great variety of intermediate degrees, which cannot well be all enumerated, and which do not call for it, as they are differences only in the amount, and not in the nature, of the alterations.

In 292 cases, Dr. Frerichs found 133 in the third stage, or almost one-half of the whole number.

Our author believes that six of the forms described by authorities so high as Bright, Rayer, Martin Solon, and Rokitansky, may be explained by the transition from the second to the third of the above stadia; these justly celebrated authors having simply described what they saw, without comparative analytical explanations.

Doubts have been raised whether the enlarged kidney of the above described second stadium is capable of passing into the atrophic form; Simon, Johnson, and others believing that the large, fatty kidney should be considered specifically distinct from the atrophied one. Simon and Johnson judge that the atrophy is produced by the distended tubes bursting, and the substances effused being afterwards removed by absorption. The possibility of this Mr. Frerichs does not undertake to deny; but he has never distinguished the anatomical traces of the occurrence.

More formidable objections have arisen to the above history of the production of new connective or cellular membrane. Henle was only satisfied that he saw it in a single case. Simon, Virchow, and others doubt it, and believe that the fibres encountered are the remains of the basal membrane of the canals, and the fibrous structure belonging to the stroma of the kidneys, rendered more compact by means of the contractions which are the necessary result of the atrophy. For the majority of cases, Dr. Frerichs acknowledges that their view is correct; but he alleges that there, notwithstanding, exist exceptions in which the new production of connective membrane cannot be doubted.

Johnson, Henle, and Simon differ from our author, in some degree, as regards the condition of the Malpighian capsules; but they also differ from one another; and the best explanation of the discrepancy is by supposing that they are all right, and that the different results which we have above enumerated occur with different degrees of frequency in particular masses of cases.

Becquerel, Rokitansky, and some others, have adhered to the idea that the granulations were enlarged capsules; but Dr. Frerichs feels supported in the preceding opinions by the general agreement with him of the English school, and cannot relinquish his convictions drawn from observation.

We have been thus minute in the general microscopic history of the disease, because it embraces the most essential portion of the whole subject, and the most important points upon which differences of opinion exist, and because it seems to us an uncommonly fine combination and conciliation of the views of the numerous and distinguished observers and writers on this novel and difficult subject. To achieve this was one of the author's principal motives, as he tells, (p. vii.,) for undertaking the task. It is sufficiently obvious that we shall have to be far more rapid in the rest of our analysis.

Several anatomical lesions are occasionally met with in the kidneys, which do not form an essential part of the disease, and yet which ought to be carefully recollected, as complicating the appearances.

Old *apoplectic caverns* are sometimes observed in the kidneys. (We greatly prefer the word *hemorrhagic* to one producing such a confusion of ideas as the above. What is the meaning of *αποπληξία*? Are the kidneys struck dumb and stupid?) Such hemorrhagic caverns are the result of an active hyperæmia at the commencement of the disease, or of febrile excitements during its course. They are broadish or irregular; from the size of a poppy-seed to that of a pea, or larger, and filled with a black or ochre-yellow matter, according to their age. They are generally found in the cortical portion, and sometimes just under the capsular membrane. Very rare cases have been found, of a wedge-shaped coagulum, with its base at the surface of the kidney, and penetrating deeply, resembling similar coagula found in the spleen.

In only six cases among 292, was the exudation changed, in particular localities, into *pus*. This generally occurred in minute specks, from the size of a pin's head to that of a lentil. In Cormack's case, one mass was as large as a walnut. The pus is, in general, thick, and surrounded by a surface of unaltered fibrinous matter.

*Cysts* were found containing a fluid approaching, in composition, to the serum of the blood; and, in Dr. Johnson's cases, fatty matter. As far as Dr. Frerichs's researches have extended, they do not contain urinary substances. They are of sizes from that of a millet-seed to that of a hazelnut or larger, and generally project from the surface, becoming conspicuous on the removal of the capsule of the kidneys. They appear to be portions of the tubules, separated from the rest by obstruction or pressure. The gelatinous contents which are found in some of these offer the reaction of mucus, and are probably obtained from the dissolution of epithelial scales. A question whether these cysts were portions of urinary tubes or Malpighian capsules, has lost its interest since the continuity of these parts has been generally acknowledged. If they, in particular instances, contain a Malpighian capsule, the coil of vessels cannot exist long, but must soon become effaced under the pressure of the distending fluid. Dr. Frerichs thinks that the fatty matter found in them by Dr. Johnson may be ascribed to the same source which supplies it to those portions of the tubes which are not separated into cysts: (formation of fat, in drops, in the decomposing epithelial cells, as described above.)

Urinary sand is frequently found in the kidneys. Urates have been met with in the pyramids and in the cortical tubes; sometimes immersed in fibrinous coagula, sometimes disseminated through the cortical matter. Rayer calls nephritis, accompanied by these, gouty nephritis. Johnson saw oxalate of lime in crystals inclosed in cells. Simon found cystin in a cyst.

Tuberculosis has been found combined with Bright's disease, in the same kidney, and Dr. Frerichs can imagine that the irritation produced by the presence of tubercular matter may have contributed to develop the other affection. Diseases of the renal vessels have been also thought capable of contributing to its production. So may tuberculosis of the lymphatic glands in the hilus of the kidney; the tumors in this case acting as a mechanical impediment to the circulation of the blood. Dr. Frerichs has seen three cases of tubercles producing retro-peritoneal abscess, which exerted an unsuspected influence in developing the malady which is the subject of our consideration. In general, however, the latter is found existing alone.

A mode of checking and confirming the observations made with so much care and detail, by the microscope, embracing the apparent effect of chemical agents under that instrument, has been sought by a chemical investigation on the larger and more familiar scale. By these means a feeling of greater certainty is given to the mind, and to this is added the kind of accuracy which is drawn from arithmetical calculation. Specimens of the cortical matter were those compared. The most striking differences were in the quantity of fat, and these seem to depend upon the degeneration and discharge of the epithelium on which we have dwelt at so much length.

In the healthy kidney, Dr. Frerichs found from 16·30 to 18 per cent. of dry, solid matter; and of the dry substance itself, 4·4 to 5·05 of a buttery fat.

In the first stadium of Bright's disease the amount of fat seems rather diminished. Of the whole substance, he found 18·14 of solid matter, and ·79 of fat; and of the solid matter, 4·35 in the hundred.

In another case, of the solid matter, 4·10 of fat.

In the second stadium, the total amount was doubled or tripled.

In one case were found 18·4 of solid matter and 1·72 of fat; and in the solid matter, when dried, 9·4 of fat.

In another case, 17·17 of solid matter, and 2·6 of fat; and of the dried matter, 13·9 of fat.

In the third stage, the ratio of fat is sometimes greater, sometimes smaller, according to the proportion formed and that evacuated.

One case furnished 15·6 of solid matter and 1·6 of fat; and of the solid matter, 10·4 of fat.

Another, of the solid matter, only 4·40 per cent.

In general, the fatty result turns out less than was expected from survey through the microscope; and Professor Frerichs cites this as a convincing proof that it is incorrect to consider all the rounded drops, without nuclei or granules, which are found in animal matter, under the instrument, as being fat or oil. It is a fine example of the thorough character of his investigations, and of his love of truth. In healthy and natural development of the liver, muscles, etc., and of the kidneys of dogs, cats, etc., the proportion of fat is much larger. He has found in the kidney of a cat 32·50 per cent., and in that of a dog, 27·20, both animals being sound, with no albumen in their urine; a valid, confirmatory proof, as our author thinks, that the impression of Canstatt, Johnson, and others, that Bright's disease should be considered as an idiopathic fatty degeneration of the kidneys, is incorrect.

In our notice of the historical retrospect, we omitted the views of these and other authors from want of space. We have not seen Dr. Johnson's last article, and the abstract of it in the French Archives has not left on our minds a strong impression of confutation. We deem our pages best filled with analyses of the fine work before us; though it is proper to state that the number is considerable and the character weighty of those who view Bright's disease as a mixture of several essentially different morbid changes that ought to be distinguished.

Diseased changes in other parts of the body are seldom wanting. In 99 cases the heart was hypertrophied, and among these, in 41 there was contraction or imperfection of the mitral valve, more rarely of the orifice of the aorta; 16 times atheromatous changes were present in the great arteries; in 42 the hypertrophy was uncomplicated with any of these changes.

In 175 the lungs were variously affected. Œdema of these organs occurred 75 times; in 4 cases complicated with œdema of the glottis. Sometimes acute, sometimes chronic, the œdema was principally accompanied by derangements of the structure of the mitral valves; and these were often the immediate cause of death. Pneumonia was present 27 times, generally amounting to a dense, wide-spread, lobar hepatization. In 2 instances gangrene of the lungs was found, and in 8, hemorrhagic infarction, these last cases being among those in which the valves of the heart were in a diseased condition. In 22 cases vesicular emphysema existed; and in 37, crude or softened tubercles.

The liver was diseased 46 times; with cirrhosis 26 times, with fatty degeneration 19 times, and once with carcinoma.

The spleen had undergone alteration in 30 cases; chronic tumor occurring 24 times, and acute 6; most of these last being met with in typhus fever. It is necessary to warn our American readers that the last phrase includes what we and so many admirers of the school formed after Chomel and Louis know more familiarly under the name of typhoid.

In the stomach morbid changes were found 31 times; 24 times indicating catarrhus ventriculi; most of the subjects last referred to being addicted to drinking. Three simple and four carcinomatous ulcers occurred in the pylorus. In one case the blackish or black softening of the stomach had taken place.

In the intestines were found traces of disease 61 times; of these, hyperæmia and catarrh of the mucous membrane 34 times; ulcerated follicles of the great intestine 13 times; tubercular ulcerations of Peyer's glands 12 times; typhous ("typhoid") ulcerations twice.

In the brain and spinal marrow, cerebral apoplexy 11 times. In these 11, hypertrophy of the heart occurred 8 times; atheromatous alterations of the arteries twice; and in one case no visible cause for the apoplexy was detected. Augmentation of serous fluid under the arachnoid was found 40 times; considerable in amount only 10 times. Distinctly characterized meningitis twice; and in both instances a tuberculous deposit. Tumor of the brain, apparently cancerous, occurred once.

. Other serous membranes. The dropsies connected with the disease require a more particular notice. Inflammations with fibrinous exudations were found in the serous membranes 81 times, in the pleura 35 times, in the peritoneum 33 times, in the pericardium 13 times.

Bones and joints. White swellings, with caries of the articular extremities of bones, were found 7 times, and necrosis twice.

Cutaneous sloughing was encountered 5 times; lithotomy had been performed once; and diphtheritic cystitis was found once.

We have been thus minute in the anatomical history of this disease, with a hope of usefulness. We believed it the portion most needed in the United States. The works on this subject in the English language have, in general, a tendency to the development and defense of particular views or comments; and we believed that a comparative view of the whole, at least up to a particular year, was well worthy of the humble but laborious employment of an analytical reviewer. As we have had occasion to observe, Professor Frerichs has not copied that modesty, highly unnecessary for him, and we believe equally so for most or all of the observers, which leads to the mere dry record of observations accumulated for some future induction, and refraining from general inferences in the existing state of science. Medicine, we fear, will always be imperfect; at least it is so in our time; and the wants of mankind call loudly for the application of reasoning, as well as observation, to the materials already in hand. Men are not to be allowed to die or suffer in order that a philosopher may adhere strictly to the forms of an inductive method; and we thank Mr. Frerichs, in the name of humanity, for omitting no source of useful improvement in a task so important to the welfare of our species. The vanity of belonging to a school, and the hope of distinction for originality, if merits at all, are such in a degree far inferior to that of gathering what is likely to be useful to mankind from every source, abstract or concrete.

It is evident that we shall have to be very rapid in the remainder of our survey. Simple Bright's disease is generally acute; and is occasioned by exposure to severe cold, by mechanical injuries to the lumbar region, or by the abuse of strong diuretics. It generally attacks suddenly, with some intensity; the urine at once is bloody; pain in the kidneys, sympathetic vomiting, and acute fever are seldom absent; and, under proper treatment, the case generally inclines to an early cure. Dropsy is often absent, and in general only occurs in cases produced by cold. An unfavorable result is most to be anticipated when the effusion is large, and tends to clog the urinary passages to a great extent. Uræmic coma and convulsions in that case generally precede the fatal termination. More rarely is death brought on by effusion into the lungs or serous cavities. It is under improper treatment, want of care, as when the patient is exposed to the open air at too early a period, or other imprudence, that the disease becomes chronic. The prognosis is generally favorable.

In cachectic subjects, the approaches of the malady are generally slow and concealed, occurring, in scattered instances, among persons much reduced by profuse suppurations, caries or tuberculosis. In a part of the cases these are all the causes observed; in others very moderate exposures to cold and damp are capable of bringing it on. Suffering from want of food and the other hardships of poverty, dissolute excesses, constitutional syphilis, or ill regulated mercurial courses, prepare subjects to receive this affection.

A purely cachectic case of Bright's disease often remains undiscovered, principally because the dropsy is absent, or because the latter is ascribed to the cachexy. The prognosis is very serious; and the causes of the cachexy often destroy life before the affection of the kidneys has run its time out. Where the origin of the disease is partly from external causes, there is some hope from treatment, but a definitive cure is even there very rare.

Habitual intemperance of itself does not seem often to produce this disease, but the subjects of such unwise indulgence are so frequently exposed to the other causes of the affection that the latter is by no means rare among such individuals. The approach is generally slow and unsuspected, achieving great disorganization before it attracts attention. Dropsy is, in general, strongly marked; dyspepsia, chronic vomiting, and pulmonary catarrh are present; and pneumonia is extremely frequent toward the close. It is needless to say that the prognosis is always very unfavorable.

It was not till within ten or twelve years that grounds of a fixed conviction, and consequent rational modification of the treatment, were established in regard to the production of Bright's disease in the fever which succeeds Asiatic cholera. F. Simon was aware of the existence of albumen in the urine of such in 1832; but since then the fibrinous moulds of the urinary tubules have been detected in correspondence with the rest of the phenomena. These have been found in a very large share of the subjects; Reinhardt and Leubuscher are quoted as having met with them in 28 out of 34 cases of the fever.

In the cholera typhoid, the urinary secretion is at first suppressed. After three or four days of the active stage of the fever, there is discharged a small quantity of turbid, dingy, or dark-yellow colored urine, rarely containing blood, but depositing a grayish sediment. The latter consists of amorphous mucus, epithelial scales, and fibrinous cylinders of various forms; and to these are sometimes added a precipitate, and crystals of oxalic acid. Boiling exhibits abundance of albumen. Singular coloring matters often appear in the urine; and of these it is hard to determine whether they arise from modifications of urinary coloring matter, or from the admixture of that of the bile. (See the American edition of

Lehmann, ii. 139, for *seven* hypothetical urinary coloring matters, with names assigned to them, but unproved to exist; and i. 284, for the absence of any definite account of that of the bile.) In their reaction, according to Professor Frerichs, they agree entirely with neither. Under nitric acid a reddish tinge is produced, but without the successive changes characteristic of bile pigment. Hydrochloric acid turns the reddish matter to a sky-blue, capable of continuing to exist for several days.

Professor Frerichs takes his description of the cholera typhoid from Reinhardt and Leubuscher. In the milder form, the patients, after a prolonged stage of reaction, complain of great weakness and exhaustion, having the head confused and heavy, and giddy on standing upright, and undergoing deceptions of the senses, as ringing in the ears, double vision, and the imagination of bright specks before the eyes. At the same time there is great tendency to sleep; the patient slumbering with the eyes half opened, giving rational answers when spoken to loudly, but replying peevishly, and falling off immediately to sleep. The pulse, with all this, is generally unaltered, in frequency, but is sometimes a little more or less frequent than in health. The head is warm, circumscribed red spots are visible on the cheeks, and the conjunctiva is injected. The digestive functions are very rarely healthy; generally there is a grayish-yellow coating on the tongue, and that organ is dry in the middle; the appetite is lost, and bilious vomiting occasionally takes place. After several days a copious urinary discharge sometimes occurs, and the expelled albuminous matter and fibrinous moulds gradually disappear, with a quiet diminution of the other symptoms. If, on the other hand, the severity of the symptoms increase, we have the severer cholera typhoid. We beg leave to remind the American reader, that the word "typhoid" does not here imply any particular connection with the dothinenteric pestilence, but merely what it meant in its original employment, since misunderstood, by Professor Chomel, to wit, "resembling typhus."

In this case the nervous symptoms augment much in severity. Deep stupefaction, and, not unfrequently, quiet delirium, occur; and the patient, at first, is to be awakened only by calling to him and shaking him, to become, after an interval, entirely insensible. Local convulsions of a single extremity, or of the face, are sometimes followed by complete epileptiform attacks; and convulsions seem to be, as usual, more frequent in children than in adults. Reinhardt and Leubuscher observed trismus once, and opisthotonos once; the latter continuing for several days. The pulse is less changed, becoming weaker, smaller, and more frequent, but not reaching in these characters the excess found in a severe typhus. In some cases it is less frequent than common. In the cases which directly follow the stage of excitement, the skin, particularly of the face, is warm and turgescent; the red of the cheeks has been compared to vermilion, and the eyes become strongly injected, but the pupil commonly continues natural. Gradually the phenomena of congestion abate and the skin becomes wet with a profuse ammoniacal perspiration. It is not uncommon for the cholera exanthema to make its appearance. The coating on the tongue, at first dirty brown, becomes dry and black; and the teeth, lips, and nasal orifices are besmeared with a dark matter of a consistency resembling that of cream. Vomiting occurs at times, a bilious or mucous matter being discharged by it; and the bowels are generally regular, discharging matters of a natural appearance.

The duration of cholera typhoid is generally from two to eight days. The milder form mostly terminates in recovery; the severer is fatal in nearly two-thirds of the cases.

Recovery is rapid, and we omit the details. The most remarkable indication of the approach of death is a steadily increasing decline of the functions of the brain and spinal marrow. Few of the cases become chronic and exhibit dropsy. The relative frequency of this occurrence after cholera is not ascertained, and appears to be various. It was great in the German epidemics of 1848, 1849, and 1850. It is obvious, as is remarked by Professor Frerichs, that no just estimate can be formed of the proportion of cases which suffer under it without microscopic examination; and it will easily be seen by those who have followed our anatomical abstract, that the rapidity of the whole illness does not, in general, leave time for the production of those large external changes in the appearance of the kidneys which strike the eye, and from which we have been accustomed to take the first impression of anatomical changes in the organ. Yet, in the texture of such kidneys, he has found the largest quantities of fibrinous coagula, while the corresponding albumen occurred abundantly in the urine. He thinks that more than half of the cases of cholera typhoid which had occurred within the last few preceding years had albuminous urine. It was more frequent in Berlin than in Prague or Göttingen. Dr. Frerichs, in fine, is of opinion that the cholera typhoid stands in nearly the same relation to Bright's disease that scarlatina does; and ascribes the typhoid symptoms in both to uræmic intoxication, and its frequent or general accompaniment, the infection of the vital fluid with carbonate of ammonia produced by the decomposition of urea.

The occurrence of dropsy and very dangerous inflammations after scarlatina has received such earnest and pressing attention from physicians of high rank, and been treated with so much nonchalance by others, that we are glad to see and recapitulate what seems to serve us as a rational and scientific explanation of the apparent contradiction. To meet with one accomplished physician who expresses the greatest alarm in regard to these terrible consequences and anticipates a heavy mortality from them, and then with another of large and prolonged practical experience, who has rarely or hardly ever met with these results, affects the mind very unpleasantly. We find quite as much difference as this in the experience of European epidemics; and the key to the difficulty is thought by our author, in common with so many other high authorities, to consist very much in the relative frequency of Bright's disease in connection with the formidable exanthema in question. "Not every case of scarlatina, and not every epidemic of this exanthematic affection is equally fitted to produce Bright's disease. The kidney derangement is sometimes very frequent, sometimes rare, and sometimes altogether wanting." Wells was convinced that it was most likely to occur after a slight and incomplete eruption; and he is supported in this by P. Frank and Hamilton. Willan, Blackall, and Plenciz, on the other hand, believe the most violent form to be that which most predisposes to such results. Vieusseux saw at an early date, "what," says Mr. Frerichs, "all later physicians of extensive means of observation have found themselves forced to acknowledge," that dropsy follows scarlatina of every degree of violence, from the mildest to the severest; and that its frequency of occurrence is above all regulated by the character of the epidemic of the time. Of the causes of this variety Dr. Frerichs acknowledges the still subsisting ignorance of the profession, nor has anything in the appearance of the eruption furnished the least explanation of the subject.

In Edinburgh, in 1835 and 1836, in eight cases of scarlatina one was affected with anasarca. In Botrell's epidemic at Rennes, in 1842 and 1843, almost every case became dropsical. Roesch's epidemic, in the first half of 1842, furnished one in ten. Dr. Frerichs himself, in East Friesland, in 1843, saw one in twenty-five. In London, in 1848, in a widespread epidemic, of which one-seventh of the cases terminated in death, James Millar observed renal anasarca in the proportion of 1 in 3·33. Haidenhain, in the spring of 1846, out of twenty-six cases found twenty-one affected with dropsy and albuminuria; or 1 in 1·24.

Albuminuria, in scarlatina *without* dropsy, is far more common than has been generally supposed. James Begbie, in 1848, found it in the whole of twenty-one cases; and in two, from the commencement of the disease. Mr. Newbigging had a similar experience. Professor Frerichs, on the other hand, examined abundance of cases in which this was not to be found by the most critical care.

The secretion of albuminous matter usually begins during the stage of desquamation. Between the eighteenth and twenty-first days, the patients, previously feeling as if they were doing well, complain of languor, want of appetite, and not uncommonly of nausea, which may be aggravated so far as vomiting. At the same time the face appears puffy, and shortly after the whole body exhibits anasarca. The preliminary symptoms which we have mentioned may be wanting, and the dropsy establish itself without warning. The urine is diminished in amount, and the secretion may be absent for a whole day. Snow mentions two cases in which the urine was totally suppressed at the kidneys for forty-eight hours, and the first fluid discharged from the bladder, in quantity a few ounces, consisted almost entirely of blood. The urine is commonly of a muddy, brownish red, sometimes of a bloody red, or resembling the washings of flesh. When blood is absent, albumen and fibrous coagula are abundantly met with.

The further progress of the dropsy does not require a separate description; it may be acute or chronic. In the first the skin is hot and makes more resistance to pressure by the finger than in common anasarca; pain is produced by pressing on the region of the kidneys; the urine is small in quantity, discharged with difficulty, thick, muddy, and very rich in albumen, and the pulse and respiration are accelerated.

The complications which add to the danger of life in the dropsy which follows scarlatina, are so important as to become characteristic and require special notice, though, from their mention in regular order in their proper place, this may produce the effect of repetition. In this dropsy there is a peculiar tendency to inflammatory diseases producing effusion of coagulated matters, albuminous and otherwise. Among the most frequent and serious is pneumonia, generally lobar, but sometimes lobular, commonly beginning without being observed, accompanied early with profuse perspirations and rapidly terminating in death. They leave behind them a gray hepatization. Hamilton saw an instance in which two-thirds of both lungs became hepatized during an inflammation which endured only two days and a half. To detect these requires very careful physical exploration. It is important to attend to the slightest difficulty of breathing in children; and it is by no means uncommon to discover by this means the existence of the disease of the kidneys where it was otherwise unsuspected.

Bronchitis is less frequent, although this too may be rapidly mortal; nor is the danger confined to children or to the tendency to be complicated with lobular pneumonia. Dr. Frerichs saw the case of a soldier, twenty-two years of age, who died within twenty-four hours after he was removed from his cantonment to the hospital, and in whom he found the mucous membrane, from the glottis to the finest bronchiæ, coated with a false membrane. This, where examined, was gray, and a quarter of a line in thickness.* Dr. Frerichs does not employ the term diphtheritis, now so familiar to us in America, in speaking of this case; nor does he, in this connection, mention œdema of the glottis. Œdema of the lungs, he says, is frequently combined with Hamilton's watery effusion, and may become dangerous in a short time. Pleurisy and pleuro-pneumonia are not uncommon after scarlatina. Pericarditis was often seen under these circumstances by Willis, but is more rarely found so than most other inflammatory affections of the chest. It is observed, contrary to what might be naturally expected, to occur much more frequently after scarlatina without albuminous urine. This is based on the cases published by Burrow, Copland, Puchelt, Rilliet and Barthez, and others, with the negative instances of Willis. Peritonitis after scarlatina is quite rare, notwithstanding the effusion of serum into the abdominal cavity. All these inflammations are also observed in the early stage of scarlatina; not necessarily depending on the previous development of Bright's disease. They are then more easily combated.

Uræmic intoxication may exist sufficiently to threaten the life of the patient, often extending to the production of blindness. Violent vomiting and profuse diarrhœa may produce serious and dangerous exhaustion.

The chronic form of dropsy after scarlatina is less important. It may continue for months, and terminate only with the Bright's disease which produced it.

The necessity of abridgment, which will compel us to omit most of the detailed comments on the very important subject of the treatment, with other valuable considerations, obliges us, after giving the above account of the anatomical appearances, nature of the disease, and symptoms, to content ourselves, during the remainder of our task, with mere occasional notices of what has struck our attention.

Albuminous nephritis may follow scarlatina without dropsy, and may prove fatal. Encephalitis, meningitis and the uræmic intoxication which resembles them, may prove fatal in the cases that follow scarlatina. A dropsy without albuminuria also occurs, (sixty cases observed by Philipp, in Berlin, in 1840,) and is generally cured. It is ascribed principally to colds, caught during or after the exfoliation of the cuticle.

Bright's disease after scarlatina is considered one of the favorable forms. Complete cures are frequently met with. They are announced, in the usual course of things, by an increase of the perspiration and urinary secretion. The unfavorable prognosis is principally to be derived from

* There is some oversight in language here. How could the finest bronchiæ contain such a thickness? or how could life continue till the completion of such an investment?

the complications which we have enumerated; inflammations, the larger watery effusions, œdema of the glottis, and exhaustion by vomiting and purging.

The disease has been observed, in a few cases, after measles and small-pox. Instances are found after typhus fever; and this is the least understood of all the forms known. Dr. Frerichs formally commits it to future laborious and extensive inquiry; and we do not see that any abstract of the facts which he has been able to collect, alleging, as he does, their scattered, incomplete, and uncertain character, and the smallness of their numbers, would be profitable to our present purpose. For a different reason we cannot abridge the important chapter on Bright's disease during pregnancy; of analyzing which the difficulty arises from its great extent of inquiry, the value of its conclusions, and the impossibility of doing justice to much of it by endeavoring to shorten it. This affection, as may well be imagined, is truly formidable, from its direct fatality, and from its frequent production of puerperal convulsions, death of the fœtus, abortion, peritonitis, pneumonia and internal metritis. Devilliers and Regnault lost 11 cases out of 20; Lever, 2 out of 12; and our author, 3 out of 7.

During the regular and direct treatment of Bright's disease, venesection, with a conscientious caution and a reasonable discretion, continues to be recommended. This is not one of the cases so much thrust before the popular eye, and not always with honorable motives, where modern advances in pathology have tended to limit the use of blood-letting. In the case of Blackall's work, modern advances in pathology, as we have seen, and as many recollect, pretty effectually extended its employment in dropsies, in which it had been scarce used at all. It is obvious to all who really desire to execute their duty, that the proper rules for the use of bleeding should be a knowledge of the patient's condition, scrupulous care, moderation and conscientiousness, and an avoidance of being governed by excitements of the mind, either from admiration of heroics, endeavors to make revolutions out of dissections or microscopic investigations, or ignorant clamor from practitioners without or within the profession. Local bleeding and counter-irritations are very much left to the authority of a few writers, and to the judgment of the practitioner in the case. A favorable account is given of purgation, a practice extensively known of old in America as the best remedy for dropsy, the success of which greatly exceeded that by diuretics.

One of the qualities for which we have felt most inclined to praise the few German inquirers into whose works we have looked, is the extreme modesty and gentleness with which they comment upon the experience of living or recent practitioners. They are, in general, well versed in the history of medicine; and few remarks more obviously result from that history than that well-informed, observing, and conscientious men in different periods of the world, and at times when very different notions prevail, will, to a surprising extent, come to the same conclusions in practice. We shall see the value of this purgative treatment again adverted to under the treatment of uræmia, and confirmed by another observer of character. The makers of profound researches in German medicine seem very often more inclined to make their discoveries agree with the convictions of those engaged in collateral labors than to gain glory by overturning them, even though it may be proper to modify them. Liebig, in his most original researches, asks his therapeutic facts from an eminent practitioner, as Hufeland, and betrays no inclination to meddle with their validity; and Dr. Frerichs, able, learned, and original as he is, never seeks to destroy the authority of others who deserve it. It is evidently the doctrine of such a school that a well-educated physician will rather seek to build up science than to overturn it, and to link in every novelty, as far as possible, with the past, by a studious and just comparison.

Uva ursi and the pyrola umbellata of our woods here retain their character, and are viewed as diuretics independently of astringency and the local anodyne effect ascribed here to their use. So does horse-radish. It is not surprising that stimulating diuretics, as cantharides, and including turpentine, are prohibited, nor does the writer of this review feel surprised or displeased that this extends to nauseants, such as squills. Dr. Frerichs thinks that, by the use of matters containing tannin in both forms, or of the gallic or tannic acids themselves, combined with aloes, he has succeeded in diminishing the albuminous admixture in the urine. Nutritious diet, and a little light wine, are advised in the anæmic condition. Warm dresses are recommended for healthy diaphoresis; but not diaphoretic drugs. Cream of tartar and digitalis are not forbidden to competent hands; and Seltzer water is included as a diuretic, to be given mixed with other diuretic drinks, or, according to the case, with wine.

The secondary inflammations are, as might be expected, pronounced difficult to manage. We have spoken of their great tendency to increase the mortality, and we shall not attempt to abridge the treatment; which requires so much limitation, and for which the author gives us so little encouragement.

Uræmic intoxication has for its most proper cure, where practicable, the restoration of the urinary secretion. This may be attempted by means of the milder diuretics which we have above enumerated. Hydragogue cathartics, as gamboge, now and then succeed where these have failed; and we observe, in a late number of the Archives, that this practice is confirmed on the authority of Professor Treitz, of Prague. It is not confined to one or a few physicians to see that much, if not nearly all of the urea in the blood, is by this time decomposed into carbonate of ammonia, of which the patient smells, and which is discharged, as remarked, we are told, by the last-named teacher, through all the emunctories. The alimentary canal naturally evacuates so large an amount of the materials of this carbonate, that there can be no doubt of the propriety of the advice to use it as the most powerful instrument of derivation.

The danger of delay is here important in view of the risk of life; and, as Professor Frerichs remarks, is more so than the accidental contra-indications.

In the last stage, where uræmia has been gradually produced, no benefit is to be expected from diuretics.

The second task, in the uræmic poisoning, is to endeavor to diminish the action of the deleterious matters upon the nervous centres. Chlorine and the vegetable acids are here proper, and these last should be varied, as by the use of benzoic acid. Bathing with vinegar, and injections containing the same acid, are here proper, and of some use. Congestion of the brain when present, may be met, as far as practicable, with alvine derivations, and, where really necessary, by local or even general detractions of blood. In cases which have made much progress, it is obvious that no permanent relief is to be expected.

Obstinate vomiting has been palliated by antiphlogistic agents, the use of small quantities of opiates, and, according to Dr. Christison, of creosote.

